# Impact of Instructions on the Positivity Effect in Younger and Older Adults’ Autobiographical Memories

**DOI:** 10.1093/geroni/igaa057.1623

**Published:** 2020-12-16

**Authors:** Karen Siedlecki, Jillian Minahan, Neshat Yazdani

**Affiliations:** Fordham University, Bronx, New York, United States

## Abstract

The positivity effect (PE) refers to age-related enhancement in memory for positive information or decreased memory for negative information. The PE is thought to be the result of age-associated shifts in motivational goals towards enhancing well-being. Research has demonstrated the PE in laboratory settings, and a meta-analysis showed that the PE was most robust in conditions with fewer instructions/constraints (Reed et al., 2014). Findings related to the PE in autobiographical memory (AM) are mixed. Goals of the current study include examining 1) whether the PE was evident across four increasingly restrictive AM conditions (involuntary AMs, voluntary AM with no constraints, voluntary AM within a specific time frame, voluntary AM within a specific time frame and related to a specific cue word), and 2) whether available cognitive resources (assessed with the Stroop task and a trail-making test) influence the PE, since cognitive control is essential for achieving goals. Participants (N=126) completed the shortened Memory Experiences Questionnaire for each memory, which assesses several phenomenological characteristics, including memory valence. There were no significant differences in memory valence between the younger (n=69, ages 18-34) and older adult (n=57; ages 60-85) groups for involuntary AMs or the less constrained voluntary AMs, but there was a significant difference for the most constrained AM wherein older adults rated their AMs as more positive than younger adults. Performance on cognitive control measures did not influence the AM valence. These results highlight the importance of considering the effects of type of stimuli and instructions when studying the PE.

